# Changes in heart rate variability over time from symptom onset of transient global amnesia

**DOI:** 10.1038/s41598-024-57546-5

**Published:** 2024-03-23

**Authors:** Soomi Cho, Sue Hyun Lee, Hye Jeong Lee, Min Kyung Chu, Won-Joo Kim, Kyoung Heo, Kyung Min Kim

**Affiliations:** 1 Department of Neurology, Severance Hospital, Yonsei University College of Medicine, 50-1 Yonsei-ro, Seodaemun-gu, Seoul, 03722 Republic of Korea; 2 Department of Neurology, Wonju Severance Christian Hospital, Yonsei University Wonju College of Medicine, Wonju, Republic of Korea; 3 Department of Neurology, Gwangmyeong Hospital, Chung-Ang University College of Medicine, Gwangmyeong, Republic of Korea; 4 Department of Neurology, Gangnam Severance Hospital, Yonsei University College of Medicine, Seoul, Republic of Korea

**Keywords:** Neurology, Neurological disorders

## Abstract

Transient global amnesia (TGA) often involves precipitating events associated with changes in autonomic nervous system (ANS), and heart rate variability (HRV) reflects the ANS state. This study aimed to investigate HRV changes after TGA. A retrospective analysis of HRV included patients diagnosed with TGA between January 2015 and May 2020. The time and frequency domains of HRV were compared among three groups: early (< 1 week after TGA, n = 19), late (1–4 weeks after TGA, n = 38), and healthy control (HC, n = 19). The Pearson’s correlation between time and time-domain HRV was also examined. The standard deviation of NN intervals (SD_NN_) (early, 47.2; late, 35.5; HC, 41.5; *p* = 0.033) and root mean square of successive RR interval differences (RMSSD) (early, 38.5; late, 21.3; HC, 31.0; *p* = 0.006) differed significantly among the three groups. Post-hoc analysis showed statistically significant differences only in the early and late groups in both SDNN (*p* = 0.032) and RMSSD (*p* = 0.006) values. However, the frequency domain with total power, low-frequency and high-frequency powers, and low-frequency/high-frequency ratio did not differ. SD_NN_ (Pearson correlation coefficient =− 0.396, *p* = 0.002) and RMSSD (Pearson correlation coefficient =− 0.406, *p* = 0.002) were negatively correlated with time after TGA. Changes in HRV occurred over time after the onset of TGA, with the pattern showing an increase in the first week and then a decrease within 4 weeks.

## Introduction

Transient global amnesia (TGA) is a clinical syndrome characterized by sudden and severe memory impairment without other neurological deficits, followed by full recovery within 24 hours^1^. Reversible selective memory disturbance is a key characteristic of TGA^2,3^. This unique and striking feature has long attracted the attention of researchers. Several pathogenic hypotheses have been proposed, including arterial ischemia, venous flow abnormality, seizure, migraine, and psychogenic disorders^4,5^. However, the pathogenesis of TGA has not been precisely elucidated.

TGA often follows precipitating events such as the Valsalva maneuver, emotional stress, a hot bath, a cold swim, physical exertion, sexual intercourse, and defecation^6,7^, most of which are closely related to changes in the autonomic nervous system (ANS), suggesting an association between TGA and ANS dysregulation. Heart rate variability (HRV) quantifies the variation in R-wave to R-wave (RR) intervals and its expression through several related variables^8–10^. HRV is considered a useful non-invasive tool for assessing ANS, particularly cardiac parasympathetic modulation^11,12^. HRV is easily measured, especially in the clinical setting where patients with TGA are first encountered and has been measured in other neurological conditions known to share pathomechanisms with TGA. While HRV has been studied in patients with potential etiologies of TGA, including cerebral ischemia, epilepsy, and migraine^13–16^, analytical studies of HRV in patients diagnosed with TGA are lacking.

Here, we aimed to identify changes in HRV in TGA patients and to determine how HRV changes over time in TGA patients compared to that in healthy controls (HCs). In addition, we wanted to identify the types of HRV that can be used to differentiate the disease clinically and explore whether they can help explain the pathogenesis of the disease.

## Results

### Clinical characteristics

The clinical characteristics of the early, late, and HC groups are shown in Table 1. No significant differences were found in the proportion of women (early, 63.2%; late, 73.7%; HC, 63.2%; *p* = 0.614) and mean age (early, 59.6 ± 8.1 years; late, 61.4 ± 7.3 years; HC, 54.4 ± 16.6 years; *p* = 0.067) among the three groups. The Mini-Mental State Examination (MMSE) scores of patients with TGA did not differ between the early and late groups (early, 24.9 ± 3.3; late, 25.0 ± 4.4; *p* = 0.960). Magnetic resonance imaging (MRI) abnormalities were identified in five patients each in the early (5/19, 26.3%) and late (5/38, 13.2%) groups (*p* = 0.275). All MRI abnormalities were dot-like diffusion-restricted lesions in the hippocampus. An electroencephalogram (EEG) showed left temporal sharp waves in one patient in the late group (1/38, 2.6%).

**Table 1:** Demographic and clinical characteristics of study participants.

	HC (n = 19)	Early (n = 19)	Late (n = 38)	*p*-value
Sex, female, n (%)	12/19 (63.2)	12/19 (63.2)	28/38 (73.7)	0.614
Age, mean (SD)	54.4 (16.6)	59.6 (8.1)	61.4 (7.3)	0.067
MMSE, mean (SD)	–	24.9 (3.3)	25.0 (4.4)	0.960
MRI, abnormal*, n (%)	–	5 (26.3)	5 (13.2)	0.275
Left hippocampus		3	0	
Right hippocampus		2	3	
Both sides of the hippocampus		0	2	
EEG, abnormal, n (%)	–	0 (0.0)	1 (2.6)	1.000

Early: patients who underwent ECG within 1 week of TGA.

Late: patients who underwent ECG between 1 and 4 weeks after TGA.

*All MRI abnormalities were dot-like diffusion-restricted lesions in the hippocampus, and the EEG abnormality was a left temporal sharp wave.

*ECG* Electrocardiogram, *EEG* electroencephalogram, *HC* healthy control, *MMSE* Mini-Mental State Examination, *MRI* magnetic resonance imaging, *TGA* transient global amnesia.

### HRV-time domain

The HRV values for each group are shown in Table 2. The mean values of RR intervals, standard deviation of NN intervals (SD_NN_), and root mean square of successive RR interval differences (RMSSD) for each group were compared in the time-domain analysis. SD_NN_ (early, 47.2 ± 19.2; late, 35.5 ± 10.9; HC, 41.5 ± 20.1; *p* = 0.033; partial eta squared (η^2^_p_) = 0.089) and RMSSD (early, 38.5 ± 29.3; late, 21.3 ± 11.0; HC, 31.0 ± 19.6; *p* = 0.006; η^2^_p_ = 0.130), which reflect the total variability and parasympathetic activity, respectively, were significantly different among the three groups. The post hoc pairwise comparison showed higher SD_NN_ (*p* = 0.032) and RMSSD (*p* = 0.006) values in the early group than in the late group. However, no significant difference was observed in the RR intervals among the three groups (early, 935.9 ± 184.2; late, 890.0 ± 114.7; HC, 899.0 ± 152.7; *p* = 0.522).

**Table 2:** Heart rate variability in patients with transient global amnesia and controls 1 week and 4 weeks after symptom onset.

	HC (n = 19)	Early (n = 19)	Late (n = 38)	*p*-value	Post hoc
Time domain					
RR intervals, ms, mean (SD)	899.0 (152.7)	935.9 (184.2)	890.0 (114.7)	0.522	
SD_NN,_ ms, mean (SD)	41.5 (20.1)	47.2 (19.2)	35.5 (10.9)	0.033*	H-E, *p* = 0.872
					E-L, *p* = 0.032*
					H-L, *p* = 0.538
RMSSD, ms, mean (SD)	31.0 (19.6)	38.5 (29.3)	21.3 (11.0)	0.006*	H-E, *p* = 0.683
					E-L, *p* = 0.006*
					H-L, *p* = 0.224
Frequency domain					
Total power, ms^2^, mean (SD)	1357.1 (1147.1)	1713.0 (1038.6)	962.3 (729.4)		
Total power, ln(ms^2^), mean (SD)	6.8 (1.0)	7.1 (1.0)	6.6 (0.8)	0.162	
LF power, ms^2^, mean (SD)	330.2 (397.8)	302.4 (193.9)	190.7 (164.4)		
LF power, ln(ms^2^) , mean (SD)	5.2 (1.2)	5.4 (1.0)	4.9 (1.0)	0.120	
HF power, ms^2^, mean (SD)	300.8 (333.4)	332.3 (321.7)	144.5 (140.9)		
HF power, ln(ms^2^) , mean (SD)	5.0 (1.3)	5.1 (1.5)	4.5 (1.1)	0.122	
LF/HF, mean (SD)	1.4 (0.8)	1.8 (1.4)	2.0 (1.6)	0.372	

Early: patients who underwent ECG within 1 week of TGA.

Late: patients who underwent ECG between 1 and 4 weeks after TGA.

The post hoc analysis employed the Bonferroni method for statistical comparisons (H-E, HC vs. Early; E-L, Early vs. Late; H–L, HC vs. Late).

*ECG* electrocardiogram, *HC* healthy control, *HF* high-frequency power, *LF* low-frequency power, *LF/HF* low- to high-frequency power ratio, *RMSSD* root mean square of successive RR interval differences, *RR* R-wave to R-wave, *SD*_*NN*_ standard deviation of N–N intervals.

*p < 0.05.

### HRV-frequency domain

In the frequency-domain analysis, we compared the natural logarithms of the total power, low-frequency (LF) power, high-frequency (HF) power, and the original values of the low- to high-frequency power ratio (LF/HF). The natural logarithms of the total power (early, 7.1 ± 1.0; late, 6.6 ± 0.8; HC, 6.8 ± 1.0; *p* = 0.162), LF power (early, 5.4 ± 1.0; late, 4.9 ± 1.0; HC, 5.2 ± 1.2; *p* = 0.120), and HF power (early, 5.1 ± 1.5; late, 4.5 ± 1.1; HC, 5.0 ± 1.3; *p* = 0.122) demonstrated no significant differences among the three groups. Additionally, no significant difference was observed in the LF/HF (early, 1.8 ± 1.4; late, 2.0 ± 1.6; HC, 1.4 ± 0.8; *p* = 0.372).

### Changes in HRV over time after TGA onset

**Figure 1:**
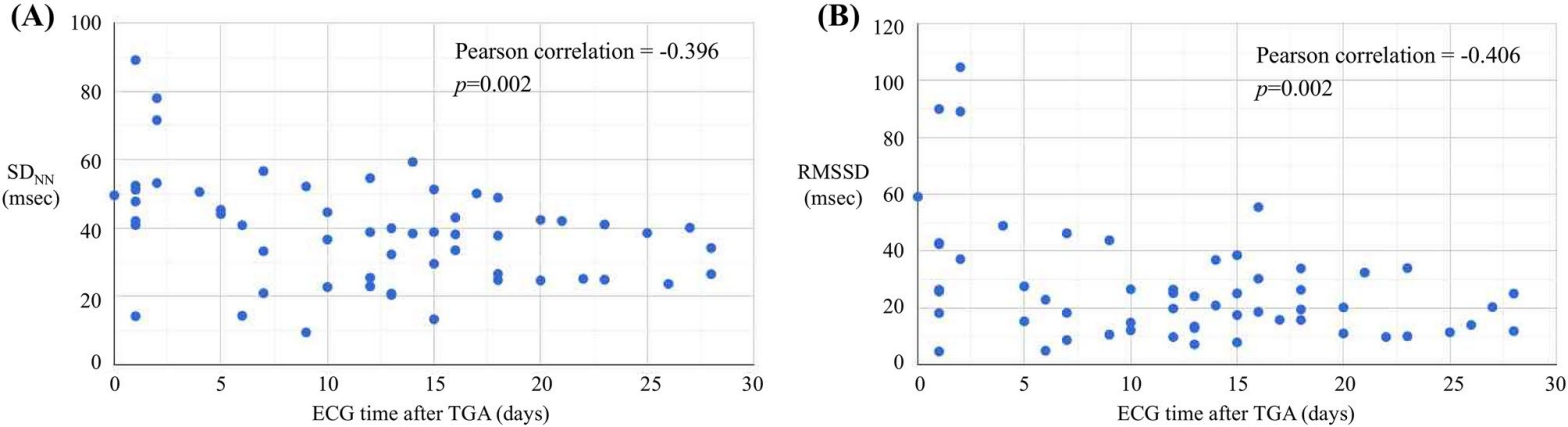
Changes in HRV—(**A**) SD_NN_, (**B**) RMSSD—after TGA. TGA, transient global amnesia; RMSSD, root mean square of successive RR interval differences; SD_NN,_ standard deviation of N–N intervals; HRV, heart rate variability.

We used the Pearson’s correlation coefficient (r) to examine the changes in SD_NN_ and RMSSD values with time intervals after TGA (Fig. 1). SD_NN_ (r = − 0.396, *p* = 0.002) and RMSSD (r = − 0.406, *p* = 0.002) had a weak negative correlation with the time interval after TGA. We also found that SD_NN_ (β = − 0.644, *p* = 0.005) and RMSSD (β = − 0.951, *p* = 0.004) showed a significant negative correlation with time intervals after TGA using a linear regression adjusted for age and sex.

## Discussion

We retrospectively assessed HRV in patients with TGA at two-time points and compared their HRV findings with those of HCs. The time-domain HRVs, SD_NN_ and RMSSD, were lower when measured between 1 and 4 weeks after TGA than when measured within 1 week after TGA. SD_NN_ and RMSSD decreased with the elapsed time after TGA. HRV measured within 1 week or between 1 and 4 weeks of TGA onset did not differ in the time or frequency domain compared to that of HC.

Three main hypotheses regarding the pathogenesis of TGA are cerebral ischemia, seizures, and migraine^5^. While no previous studies have directly explored the link between TGA and HRV, alterations in HRV associated with cerebral ischemia, seizures, and migraines have been documented. Prior research has reported reduced HRV in patients with cerebral infarction^17,18^, epilepsy^15,19,20^, and migraines^21,22^. In particular, HRV was not higher in patients within hours or days of symptom attack of cerebral ischemia, seizures, or migraine than in healthy controls. The higher HRV observed in our study within 1 week after TGA suggests that other pathomechanisms than those commonly proposed may underlie TGA. The higher HRV observed in the present study within 1 week after TGA leads us to expect a role for HRV as an adjunct to differential diagnosis in situations where it is clinically necessary to differentiate from stroke, epilepsy, and migraine, and further suggests that pathologic mechanisms other than those commonly suggested may underlie TGA. This is also consistent with the inability of any of these three hypothesized mechanisms to fully explain the pathogenesis of TGA.

In this study, we observed a temporary increase in HRV during the first week following a TGA episode, a pattern consistent with prior research revealing alterations in physiological indicators during the same timeframe. Among patients who underwent single-photon emission computed tomography within the initial week post-TGA, researchers detected a transitory reduction in perfusion, notably in the left hippocampus and left thalamus^21^. Additionally, an examination of EEG power spectra within the first week following a TGA episode revealed significantly diminished absolute beta 1 (12–17.9 Hz) and alpha power in TGA patients compared to those in HCs. This decrease was particularly pronounced in the left-sided locations and left temporal location^23^. These findings collectively suggest that the physiological changes occurring within the initial week following a TGA episode may have relevance to the condition. Up to 90% of patients experience pre-TGA onset precipitating events^24,25^. Women and men are more likely to experience emotional and physical precipitating events, respectively^26^. Events preceding TGA, such as emotional stress, bouts of exercise, and Valsalva maneuvers, are commonly accompanied by changes in the state of ANS. In previous studies, HRV decreased immediately after emotional stress in short experimental settings lasting less than 30 min before returning to baseline levels^27–29^. Additionally, HRV parameters demonstrate a curvilinear decline during exercise, a time-dependent recovery upon exercise cessation, and an eventual return to pre-exercise levels within minutes or up to 72 hours^10,30–33^. Notably, during post-exercise recovery, an HRV rebound phenomenon was observed, during which HRV overshoots above the pre-exercise levels in the hours or days after short and long exercise periods; however, the precise underlying mechanism is unclear^31,34,35^. In this study, HRV was elevated within 1 week after TGA and then decreased to a level not significantly different from that of controls. Although more data are needed, we speculate that the transient elevation may be a rebound phenomenon.

We observed decrease patterns in both the SD_NN_ and RMSSD as time progressed following a TGA episode. These findings are significant as SD_NN_ and RMSSD are key indicators of HRV, reflecting different aspects of ANS function. Specifically, SD_NN_ represents overall HRV and is considered a marker of both sympathetic and parasympathetic activity, while RMSSD primarily reflects parasympathetic (vagal) activity, which is linked to heart rate deceleration^36^. The autonomic adjustment observed suggests a phase of recovery from the acute stress of TGA, where the initial heightened autonomic activity diminishes as the body returns to a stable state^37^. This reflects the ANS’s dynamic response after TGA, highlighting the usefulness of HRV measurements as indicators for recovery tracking and understanding TGA's autonomic effects.

This study has several limitations. First, continuous changes in HRV over time after TGA could not be identified, as HRV was not continuously measured. Second, HRV, which can be influenced by circadian rhythm, was measured in this study within the range of 9 AM to 5 PM. It is worth noting that the timing of measurements could introduce some variability as a potential limitation of the study. Third, we used a single-center design, and the study had a small sample size. Fourth, whether the transient elevation in HRV was primary or a consequence of TGA is unknown. In a clinical setting, however, it is nearly impossible to predict the moment TGA occurs and measure HRV just before the symptom onset of TGA.

This study also has several strengths. First, we determined the changes in HRV after TGA by reviewing electrocardiograms (ECGs) at two time points. To the best of our knowledge, this is the first study to describe post-TGA HRV changes over time. Second, we suggested the use of HRV for future research on TGA. Measuring HRV in TGA patients is readily accessible because ECG is commonly performed with EEG, which is frequently used for the differential diagnosis of TGA. Third, we made notable efforts to accurately diagnose TGA, which is defined by diagnostic criteria based primarily on patient- and witness-reported symptoms. Neurologists performed the clinical diagnoses of TGA in our study, and differential diagnoses were ruled out based on clinical, laboratory, radiological, and electrophysiological findings.

In conclusion, we found that TGA and changes in the state of ANS were related and that post-TGA HRV changed over time, showing an increase during the first week followed by a decrease within 4 weeks. Further studies with larger samples and continuous HRV monitoring may provide a deeper understanding of the role of temporary autonomic dysfunction in TGA, which may shed light on the pathogenesis of TGA.

## Methods

### Data and participants

We created a study design and collected data for a retrospective observational study comparing HRV in patients with TGA and HCs. We retrospectively reviewed the data of 684 consecutive patients who visited the emergency department of Yongin Severance Hospital in Seoul, Republic of Korea, between January 2015 and May 2020 and were diagnosed with TGA. Neurology residents first examined all patients in the emergency department. A neurologist (KMK) then confirmed the diagnosis on the basis of medical records according to the Hodges and Warlow criteria^1^. Demographic and clinical characteristics, MMSE scores, brain MRI, and ECG with EEG data were collected. We excluded patients without MRI or ECG findings. Patients who underwent ECGs more than 4 weeks after the TGA episode were also excluded.

**Figure 2:**
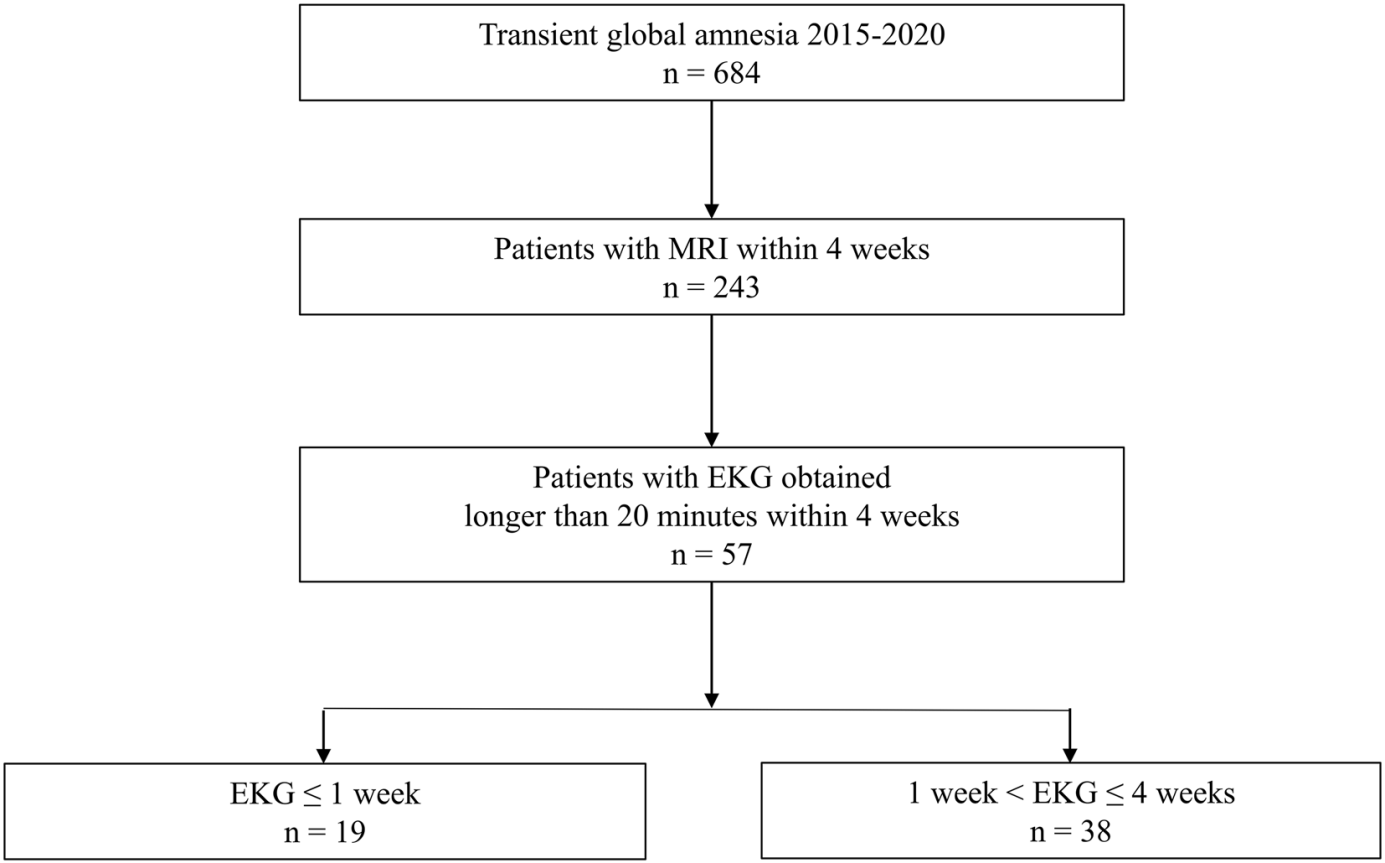
Flowchart of participant enrolment.

Of the 684 patients, 243 underwent MRI within 4 weeks, and only 57 underwent MRI and ECG over 20 min within 4 weeks of TGA onset. Of the 57 patients with TGA finally enrolled, those who underwent ECG within 1 week of TGA onset were assigned to the early group. Patients who underwent ECG between 1 and 4 weeks after the TGA episode were assigned to the late group. The selection of participants for this study is summarized in Fig. 2. ECG data were also collected from 19 HCs without medical, neurological, or psychiatric disorders who visited our institution for medical check-ups.

In the MRI analysis, abnormalities were defined as cases in which a neuroradiologist visually identified and reported any abnormality. For the EEG analysis, abnormalities were determined based on the identification of epileptiform discharges by an epileptologist.

This study was approved by the institutional review board of Yongin Severance Hospital, Yonsei University (No. 9-2020-0125). The requirement for written informed consent was waived owing to the retrospective nature of the study. Our research adhered to the ethical guidelines of the institutional and national research committees, aligning with the principles of the 1964 Declaration of Helsinki and its subsequent updates. This study followed the Strengthening the Reporting of Observational Studies in Epidemiology (STROBE) checklist.

### HRV

HRV was recorded between 9 am and 5 pm under conditions of temperature and humidity appropriate for a hospital setting. HRV was analyzed using the ECG waveform obtained simultaneously with EEG. ECG was recorded using two electrodes placed at the parasternal area in the fourth intercostal space, similar to the V1 and V2 placements in general ECG recordings with 200 Hz sampling rate. All ECGs were recorded for at least 20 min, and 20-min concatenated segments were selected for analysis. The RR files were then exported to an HRV analysis program created in MATLAB^38^. Each ECG recording was manually inspected to avoid abnormal QRS wave morphology and movement artifacts and to ensure that the R-waves were correctly marked by the program to allow accurate detection of the RR intervals. The HRV analysis program applied a linear time-domain analysis to the entire 20-min segment to measure the mean and variance of the RR intervals. The following values were obtained: the mean of all RR intervals, SD_NN_, and RMSSD. Frequency‐domain parameters were computed using fast Fourier transform based on 5‐minute epochs that were averaged and included total power, LF power (0.04–0.15 Hz), HF power (0.15–0.40 Hz), and the LF/HF. All HRV analyses adhered to the analysis methods prescribed by the HRV Task Force in 1996^39^.

### Statistical analyses

The Statistical Package for Social Sciences (SPSS v24.0; IBM, Armonk, NY, USA) was used for all statistical analyses. Student’s t-tests or analyses of variance were used for continuous variables in demographic, clinical, and HRV variables when normality was confirmed using the Kolmogorov–Smirnov test. The post-hoc analysis employed the Bonferroni method for statistical comparisons of HRV variables among groups. Chi-square tests were used to compare categorical variables in demographic and clinical variables. Pearson's correlation analysis and multiple linear regression were conducted to examine the relationship between time after symptom onset and SDNN and RMSSD. We used partial eta squared (η^2^p) to quantify the effect size. Statistical significance was set at a two-tailed *p*-value of < 0.05.

## Data Availability

The data not provided in the article may be shared upon request to any qualified investigator for the purposes of replicating the procedures and results.
