# Author Correction: Changes in heart rate variability over time from symptom onset of transient global amnesia

**DOI:** 10.1038/s41598-024-62399-z

**Published:** 2024-06-03

**Authors:** Soomi Cho, Sue Hyun Lee, Hye Jeong Lee, Min Kyung Chu, Won‑Joo Kim, Kyoung Heo, Kyung Min Kim

**Affiliations:** 1 Department of Neurology, Severance Hospital, Yonsei University College of Medicine, 50-1 Yonsei-ro, Seodaemun-gu, Seoul, 03722 Republic of Korea; 2 Department of Neurology, Wonju Severance Christian Hospital, Yonsei University Wonju College of Medicine, Wonju, Republic of Korea; 3 Department of Neurology, Gwangmyeong Hospital, Chung-Ang University College of Medicine, Gwangmyeong, Republic of Korea; 4 Department of Neurology, Gangnam Severance Hospital, Yonsei University College of Medicine, Seoul, Republic of Korea

Correction to: *Scientific Reports* 10.1038/s41598-024-57546-5, published online 23 March 2024

The original version of this Article contained an error in the Funding section. The Grant Number of the Yonsei University College of Medicine was incorrect.

“This study was supported by a research grant from Yongin Severance Hospital, Yonsei University College of Medicine, Korea. This study was supported by a faculty research grant of Yonsei University College of Medicine (1-2020-0068). The sponsors had no role in the study design; the collection, analysis, and interpretation of the data; the writing of the report; or the decision to submit this article for publication.”

now reads:

“This study was supported by a research grant from Yongin Severance Hospital, Yonsei University College of Medicine, Korea. This study was supported by a faculty research grant of Yonsei University College of Medicine (6-2020-0068). The sponsors had no role in the study design; the collection, analysis, and interpretation of the data; the writing of the report; or the decision to submit this article for publication."

The original Article has been corrected.

